# Differences in the Magnitude of Representational Momentum Between School-Aged Children and Adults as a Function of Experimental Task

**DOI:** 10.1177/2041669518791191

**Published:** 2018-08-12

**Authors:** Nobu Shirai, Erika Izumi, Tomoko Imura, Masami Ishihara, Kuniyasu Imanaka

**Affiliations:** Department of Psychology, Faculty of Humanities, Niigata University, Japan; Graduate School of Modern Society and Culture, Niigata University, Japan; Department of Psychology, Faculty of Integrated Arts and Social Sciences, Japan Women’s University, Japan; Department of Human Sciences, Tokyo Metropolitan University, Japan; Department of Health Promotion Sciences, Tokyo Metropolitan University, Japan

**Keywords:** representational momentum, development, childhood, task dependence

## Abstract

Representational momentum (RM) is the phenomenon that occurs when an object moves and then disappears, and the recalled final position of the object shifts in the direction of its motion. Some previous findings indicate that the magnitude of RM in early childhood is comparable to that in adulthood, whereas other findings suggest that the magnitude of RM is significantly greater in childhood than in adulthood. We examined whether the inconsistencies between previous studies could be explained by differences in the experimental tasks used in these studies. Futterweit and Beilin used a same-different judgment between the position where a moving stimulus disappeared and where a comparison stimulus reappeared (judging task), whereas Hubbard et al. used a task wherein a computer mouse cursor pointed to the position where the moving stimulus disappeared (pointing task). Three age groups (*M* = 7.4, 10.7, and 22.1 years, respectively) participated in both the judging and pointing tasks in the current study. A multivariate analysis of variance with the magnitudes of RM in each task as dependent variables revealed a significant main effect for age. A one-way analysis of variance performed for each of the judging and pointing tasks also indicated a significant main effect of age. However, post hoc multiple comparisons detected a significant age effect only for the pointing task. The inconsistency between the judging and pointing tasks was discussed related to the distinct effect size of the age difference in the magnitude of RM between the two tasks.

## Introduction

The perception of dynamic and directional visual patterns (visual motion) is undoubtedly important for our perception of our own body state, the environment, and the interaction between them. Various types of perception and relevant adaptive actions depend on our ability to perceive motion. These include perceiving or controlling the direction of self-motion (Warren & Hannon, 1988), intercepting a moving object (Savelsbergh, Whiting, & Bootsma, 1991), estimating the time to collision with an obstacle (Bootsma & Oudejans, 1993), perceiving individuals (Johansson, 1973), and so on.

Despite the importance of visual motion perception, our visual system faces a fundamental challenge in perceiving dynamic visual events. The visual system needs time to process the neural signals that originate from visual stimuli, so the perceived location of a moving object should lag behind its actual location at that moment in real time. However, we are apparently able to perceive the location in real time. This can be achieved by complementing the representation of a dynamic event with a *prediction* about the state of the dynamic event in the next moment. For instance, when we observe a moving object and the object suddenly disappears, we tend to make a systematic error when identifying the position where the object disappeared: The memorized point of disappearance is usually shifted in the direction of the motion of the moving object. This phenomenon is called representational momentum (RM) because the position of the moving object in our memory is shifted in the direction of motion by the momentum exhibited by our mental representation (Freyd & Finke, 1984). RM seems to be related to relatively higher (cognitive) mechanisms, rather than to lower sensory mechanisms, as the shift in position of a disappearing object depends on object’s anticipated path of motion rather than its actual path of motion (Hubbard & Bharucha, 1988). RM may share common mechanisms with other forms of *predictive* perception of dynamic events, such as the flash-lag effect (e.g., Nijhawan, 1994), the phenomenon whereby the perceived position of a stationary object often lags behind the perceived position of a moving target (for review, see Hubbard, 2014). Such apparent positional shifts of moving objects and relevant static objects may compensate for the delay incurred by the neural processing in our visual system when perceiving dynamic events (e.g., Hubbard, 2005; Nijhawan, 2002).

The *predictive* perception of dynamic events is observed at relatively early stages of life. Perry, Smith, and Hockema (2008) demonstrated that 2- to 3-year-old toddlers potentially experience RM. In their experiments, toddlers were shown a toy car moving down a slope. A barrier could be placed around the slope to stop the toy car at an appropriate position on the slope. Motion in the picture plane was from the upper left to the lower right, and the toddlers’ line of sight was perpendicular to the picture plane. An opaque occluder was set over the bottom half of the slope to prevent toddlers from directly observing the position at which the toy car stopped. However, because the barrier was higher than the occluder, the toddlers could see the position of the barrier and use this as a cue to guess the position at which the toy car would stop. There were several small doors on the surface of the occluder, from which the toddlers could retrieve the toy car. In this experimental setting, Perry et al. (2008) found that 2 to 3 year olds made a systematic error in choosing which door to open. Rather than choosing the closest door to the actual stopping point of the toy car, the toddlers chose a door farther in the direction that the toy car was moving. Perry et al. (2008) argued that the results indicated that these 2- to 3-year-old toddlers overestimated the stopping position of a moving object in its direction of motion, thus exhibiting RM.

Although several studies have confirmed that RM is also exhibited in later childhood, such as at school age, the findings are inconsistent. Futterweit and Beilin (1994) reported that the magnitude of RM was comparable in school-aged children (younger children, mean age = 8.9 years; older children, mean age = 10.9 years) and adults. They used sequences of snapshots of various actions (e.g., a person walking, running, jumping, etc*.*) as visual stimuli. In their experiment, two frames of each action sequence were presented in series. Then, participants were asked to judge whether the second frame was the same as the first one. The second frame showed a position that was either backward or forward relative to the first frame. It was expected that if the participants experienced RM, the second frame would be more likely to be considered the same as the first frame when the second frame was a forward frame. On the other hand, it was expected that participants would perceive the second frame as different from the first frame when the second frame was a backward frame. In all age groups, the *same* response was more frequently selected in the cases with the forward frames than with the backward frames. No significant differences among age groups were observed in the rate of the *same* response with the forward frames.

Another developmental study reported that the magnitude of RM decreased significantly between school age and adulthood. Hubbard, Matzenbacher, and Davis (1999) tested younger (mean age = 6.7 years) and older (mean age = 10.7 years) children and adults using visual stimuli showing *real* motion. In their experiment, a moving target appeared on a computer screen and then suddenly disappeared. The participants were required to use a mouse cursor to point to the position at which the target disappeared. The magnitude of RM was calculated by subtracting the position indicated by the mouse cursor from the position at which the target disappeared. They found that the younger (but not older) children exhibited greater RM magnitude than did the adults and concluded that the younger children exhibited greater RM than the adults.

Hubbard et al. (1999) proposed a possible interpretation for the inconsistency of these findings compared with the findings of Futterweit and Beilin (1994), which showed no significant developmental change between childhood and adulthood: Because younger children may be less sensitive to dynamic events represented by static figures than adults are, the use of static figures as visual stimuli might have reduced RM in the younger children tested by Futterweit and Beilin (1994). However, even younger children (Carello, Rosenblum, & Grosofsky, 1986; Friedman & Stevenson, 1975) and infants (Shirai & Imura, 2014, 2016) are able to perceive dynamic events from still images. Hence, the proposal offered by Hubbard et al. (1999) does not fully explain the inconsistency between their results and those of Futterweit and Beilin (1994).

Hubbard et al. (1999) discussed another explanation for the larger RM observed in younger children than in adults: Because RM relies more on an analog representation than on a propositional representation (Kelly & Freyd, 1987), younger children, who might be more dependent on analog than on propositional representation, showed greater RM than did adults. However, recent behavioral and neural research has shown that sensitivity to smooth visual motion events is significantly lower even in later childhood than in adulthood (e.g., Falkenberg, Simpson, & Dutton, 2014; Gilmore, Thomas, & Fesi, 2016; Joshi & Falkenberg, 2015). Although younger individuals may generally rely more on analog representation than older individuals do, with regard to the perception of smooth visual motion, younger individuals seem to have poorer representation of a moving object than do older individuals. Thus, the difference in the use of analog representation might not be an exclusive explanation for the larger magnitude of RM in younger individuals observed by Hubbard et al. (1999).

One novel alternative explanation for the inconsistency between the two sets of results is that they arose from differences in the experimental paradigm used to measure the magnitude of RM. Futterweit and Beilin (1994) asked the participants to judge whether two visual stimuli were the same or not; therefore, the participants were engaged in a recognition task. Conversely, Hubbard et al. (1999) asked the participants to point directly at the position at which the moving target disappeared; therefore, the participants were engaged in a recall task. Moreover, the difference between the judging and pointing tasks may be comparable to that between passive and active perceptual tasks related to separate visual systems for perception and action, such as ventral and dorsal pathways (cf. Goodale & Milner, 1992, 2004). When we observe pictorial illusion figures, such as the Ebbinghaus (Titchener) illusion, the figures produce a compelling perception of visual stimuli’s over or underestimated size; a central circle surrounded by a circular array of larger or smaller circles tends to be perceived as smaller or larger than its actual size. However, when we pick up the central circle surrounded by a circular array in an Ebbinghaus figure by the thumb and index finger, the perceived distance between them is less affected by the apparent size of the central circle and is adjusted appropriately by the actual size of the central circle (e.g., Aglioti, DeSouza, & Goodale, 1995; Haffenden & Goodale, 1998, 2000; Haffenden, Schiff, & Goodale, 2001; but see also Franz & Gegenfurtner, 2008; Pavani, Boscagli, Benvenuti, Rabuffetti, & Farnè, 1999). The difference between *seeing* and *action* in the effect of a pictorial illusion is explained by the functional difference between the two separate visual pathways; the ventral pathway mediates the perception of a visual scene and is deceived by the illusory figure, whereas the dorsal pathway mediates motor actions guided by visual information and is not deceived by the illusion. Because the judging and pointing tasks for RM are also related to *seeing and action*, respectively, it is plausible that the difference in the experimental paradigm (i.e., whether a judging or pointing task was performed) might have affected the results of the two studies. We explored this possibility by instructing the participants, who were younger children, older children, and adults, to perform both the judging and the pointing tasks. The children who participated in the current study ranged from 6 to 12 years of age. Participants were divided into two groups (younger vs. older children) to approximately match the age range with that of the previous studies (Futterweit & Beilin, 1994; Hubbard et al., 1999).

The judging task used in the current study required participants to orally judge whether the position on a computer screen at which a moving target (moving either rightward or leftward) disappeared was the same as the position at which a subsequent comparison stimulus appeared. The pointing task required participants to indicate the position at which a moving target (moving either rightward or leftward) disappeared by touching a touch-sensitive computer screen. In addition, the judging and pointing tasks each included two types of trials (immediate- and delayed vanish), which differed according to the timing of the object’s vanishing, to calculate the magnitude of the RM. In the immediate-vanish trials, a moving character vanished immediately after reaching an arbitrary position on the computer screen. Under the delayed-vanish condition, the character reached an arbitrary position, remained there for 500 ms, and then vanished. Because under the delayed-vanish condition, the moving character remained stationary for 500 ms before it disappeared, any differences between participants’ responses and the actual vanishing position could be regarded as potential response biases that were irrelevant to RM. Thus, data from the immediate and the delayed trials were used to define the magnitude of each participant’s RM by subtracting the reported displacement of the position at which the character vanished under the delayed-vanish condition from that under the immediate-vanish condition. The specific advantages of this new type of control will be discussed later.

Pointing actions could vary among the different combinations of hands used for pointing (left or right) and the direction of the moving target (leftward or rightward). For instance, when one points to a target moving toward the left or right using the right or left hand, the kinematics relevant to the pointing action involves primarily stretching the right or left elbow. On the other hand, when one points to a target moving to the right or left using the right or left hand, the relevant kinematics involves stretching and abduction of the right or left elbow. This means that different combinations of hand use and moving-target direction may result in variations in pointing actions, rendering direct comparisons among the magnitudes of RM in different age groups problematic.

For instance, participants in the current study could choose to use either the right or the left hand, and they were allowed to change hands on a trial-by-trial basis. Although we did not expect this (and thus made no video recording of the experiments), some participants, especially young children, often changed the hand used for pointing during an experimental session. This may be because children have a shorter reach than adults, which might make it easier to touch the final position of a moving target with the hand on the same side as the final position. This informal observation implies that there might be individual (and potentially age) differences in the kinematics relevant to pointing actions. Older individuals might tend to use one hand (e.g., the dominant hand) consistently across experiments; thus, the kinematics of pointing actions could vary between *stretching of the elbow and stretching and abduction of the elbow* depending on the final position of the target in each trial. In contrast, younger individuals might change the hand used for pointing according to the final position of the target (e.g., always use the right or left hand when the final position was on the right or left side of the screen). In such cases, the kinematics of pointing actions could consistently involve *stretching and abduction of the elbow* regardless of the final position of the target. These individual (and potentially age related) variations in the kinematics of pointing could act as noise during analysis of RM in the pointing task experiment according to age-group. However, it should be noted that, ideally, such noise, which is related to the kinesiology of the human body, should be equivalent under the immediate- and delayed-vanish conditions. Thus, in theory, subtracting the results under the immediate-vanish condition (RM + noise) from those under the delayed-vanish condition (non-RM + noise) should functionally cancel out any such noise.

Notably, we also adopted the subtraction paradigm in the judging task for the following two reasons. First, we wanted to analyze the results of the judging task using the same method as was used for the pointing task. Second, even in the judging task, participants might have unexpected biases in estimating the position of the vanished character (e.g., tend to overestimate or underestimate the position relative to the direction of motion of the object unrelated to RM). Such unexpected potential biases in the judging task are also ruled out by the subtraction paradigm.

## Methods

### Ethics Statement

The experimental procedures performed in this study were approved by the Ethics Committee for Psychological Research of Niigata University and were conducted according to the principles of the Declaration of Helsinki. Written informed consent was obtained from all participants (and, in the case of the children, from their parents as well).

### Participants

The final sample consisted of 16 younger children (7 females, mean age = 7.4 years, standard deviation [*SD*] = 0.7, age range = 6.7–9.1 years), 16 older children (10 females, mean age = 10.8 years, *SD* = 0.7, age range = 9.8–12.0 years), and 16 adults (8 females, mean age = 22.0 years, *SD* = 0.7, age range = 20.4–23.4 years). All participants had normal or corrected-to-normal vision and had no reported history of any visual or motoric disorder. An additional male child aged 9.9 years also took part in the experiment but was excluded from the final analysis. This child did not follow the experimental instructions and made no systematic responses to the visual stimuli.

### Apparatus

A 27-in. LCD touch-sensitive screen (ProLite T2735MSC; resolution: 1920 × 1080 pixels; refresh rate: 60 Hz; size of the presentation field: height = 336.2 mm; width = 597.6 mm; Iiyama, Inc.) was used to present visual stimuli in both the judging and pointing tasks. In addition, the touch-sensitive function of the LCD screen was used to retrieve participants’ responses in the pointing task. The presentation software package (version 17.1; Neurobehavioral Systems, Inc.) was used for stimuli presentation and to record participants’ responses. This software was run on a personal computer (CF-AX3NEABR; Panasonic, Inc.).

### Stimulus

**Figure 1.**
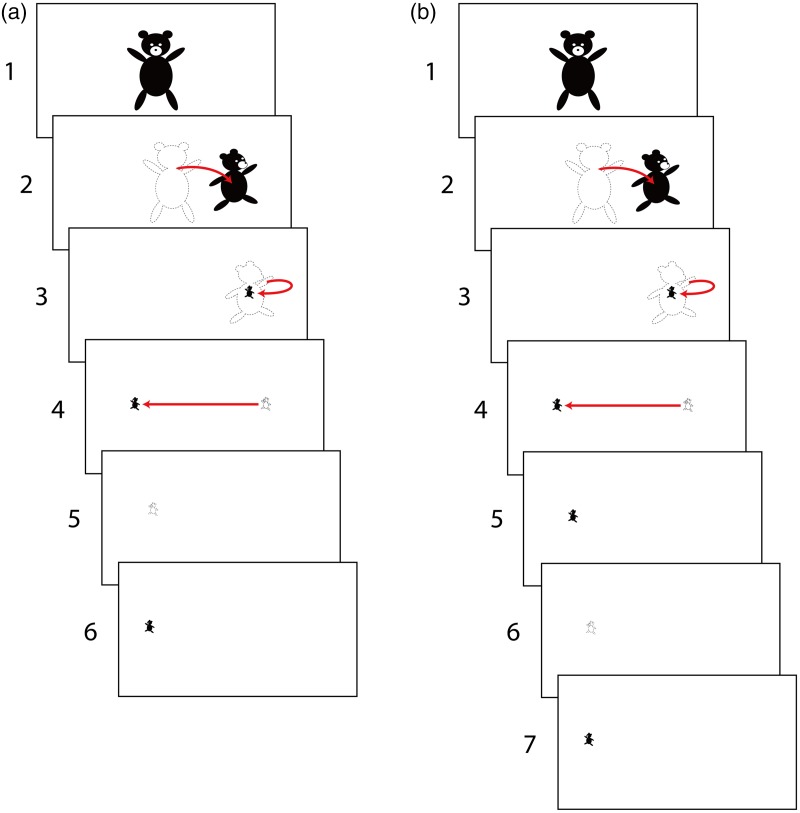
(a) Flowchart of the visual stimuli used under the immediate-vanish condition. (1) At the beginning of the trial, a cartoon character was presented at the center of the presentation field. (2) After 1900 ms, the body size of the character decreased rapidly as it jumped toward either the right or the left edge of the presentation field. (3) At a randomly chosen time between 500 and 1000 ms after Step 2, the character’s size shrank again, and (4) it began to run toward the other side of the presentation field. (5) The character vanished immediately after reaching the end position. Moreover, in the judging task, (6) after 400 ms, the character reappeared to the right or left of the position from which it vanished. (b) Flowchart of the visual stimuli under the delayed-vanish condition. (1) At the beginning of the trial, a cartoon character was presented at the center of the presentation field. (2) After 1900 ms, the body size of the character decreased rapidly as it jumped toward either the right or the left edge of the presentation field. (3) At a randomly chosen time between 500 and 1000 ms after Step 2, the character’s size shrank again, and (4) it began to run from its current position toward the other side of the presentation field. (5) The character remained at the end position for 500 ms and (6) then vanished. In the judging task, (7) after 400 ms, the character reappeared to the right or the left of the position from which it vanished (for more details, see the Stimulus and Procedure sections).

To direct the young children’s attention to the experiment, we used a moving cartoon character as the visual stimulus (Figure 1). At the beginning of each experimental trial, the character (465 pixels [14.4 deg] in width and 644 pixels [19.8 deg] in height) was presented at the center of a white presentation field. The aim of this presentation mode was to capture the participant’s attention. The character’s body was colored black. After 1900 ms, the character’s body size rapidly decreased to 390 pixels (12.1 deg) in width and 535 pixels (16.6 deg) in height, and it jumped toward a position 500 pixels (15.5 deg) away at the right or left (randomly chosen in each trial) edge of the presentation field. After a randomly chosen interval of 500 to 1000 ms, the character’s size shrunk again to a width of 78 pixels (2.4 deg) and a height of 105 pixels (3.3 deg). Then, it began to run from its current position toward the other side of the presentation field. The speed of the character increased from 0 to 30 pixels/frame (52.1 deg/s)^1^ in the first five frames, after which it remained at a constant speed of 30 pixels/frame (52.1 deg/s). The distance between the initial and end positions was randomly chosen from 960 to 1410 pixels (30.6–42.1 deg). After the character reached the final position, it behaved in accordance with the immediate-vanish or the delayed-vanish protocol. In the immediate-vanish trials, the character vanished immediately after reaching the end position. In the delayed-vanish trials, the character remained at the end position for 500 ms and then vanished.

### Procedure

Each participant sat in front of a touch-sensitive LCD screen with no supportive equipment such as a head or chin rest. One experimenter, who was naïve regarding the predictions and hypotheses of the current study, sat beside the screen and the participant. Thus, the visual stimuli on the screen were not totally invisible from the typical vantage point of the experimenter. However, because the experimenter was naïve regarding the predictions and hypotheses of the current study, possible sources of experimental bias, such as the experimenter effect, may have been minimized. The experimenter adjusted the distance between the participant and the screen to 57 cm before starting the experimental session. During the experimental session, the experimenter monitored the distance between the participant and the screen. If the viewing distance looked significantly shorter or longer than the initial distance, the experimenter gently urged the participant to return to the sitting position adopted at the beginning of each trial. Each participant took part in two experimental tasks, the judging task and the pointing task. The order of the two experimental tasks was counterbalanced across participants. The time required to complete the entire experiment, including the short rests between experimental sessions, was usually less than 20 minutes, even among younger children.

#### Judging task

In each trial of the judging task, the character reappeared to the right or left of the position from which it vanished after 400 ms. The participant’s task was to report orally whether the position of the reappeared character on the screen had shifted forward or backward in the direction of the character’s motion and relative to the position at which it vanished. The participants had three alternatives: *forward, backward, or ambiguous (I don't know)*. We used the staircase method to measure the point of subjective equality (PSE) for the judgment of the position shift of the character after it reappeared relative to the position from which it vanished. In each trial, the distance by which the character had shifted after it reappeared could increase or decrease, based on the judgment made by the participant. If a participant made a correct judgment, the absolute size of the shift decreased, while its direction (a sign of ± corresponding to forward or backward) was maintained. If a participant made an incorrect judgment (or said, *I don't know*), the absolute size of the shift increased while its direction was reversed. The initial step size of the decreasing or increasing distance by which the character had shifted when it reappeared was set to 200 pixels (6.2 deg). At every reversal point, the step size decreased by half. Once the step size reached 25 pixels (0.8 deg), it was kept fixed at 25 pixels (0.8 deg) until the end of the experiment. In the first trial of each experimental session, the position at which the character reappeared after it finally vanished was always shifted backward by 400 pixels (12.4 deg) (−400 pixels [−12.4 deg]) from the vanishing position. Each experimental session continued until the direction of the position shift had been reversed 12 times. The PSE was defined as the mean of the shift in pixels over the last 10 reversals.

The immediate- and delayed-vanish trials followed a block session design. Each participant engaged in a session with immediate-vanish trials and with delayed-vanish trials for PSE measurement. Each session typically lasted a few minutes, even among the younger children. The order of the two sessions was counterbalanced across participants. We calculated the magnitude of the RM in the judging task by subtracting the PSE under the delayed-vanish condition from that under the immediate-vanish condition.

#### Pointing task

In the pointing task, the visual stimuli were identical to those used in the judging task, except that the character did not reappear after vanishing (i.e., Frames 6 and 7 in the left and right columns of Figure 1, respectively, were not presented). The participants were instructed to touch the position where the character vanished on the touch-sensitive screen. The touched position was recorded by the touch-sensitive devices built into the screen. The displacement along the horizontal axis between the touched position and the position where the character vanished was recorded in each trial. The immediate- and delayed-vanish trials were conducted with a block session design. Each participant engaged in an experimental session of 30 immediate-vanish and 30 delayed-vanish trials, for a total of 60 trials. Each session (30 trials) typically lasted for a few minutes, even among the younger children. The order of the two experimental sessions was counterbalanced across the participants. The magnitude of RM for each participant was calculated by subtracting the mean displacement under the delayed-vanish condition from that under the immediate-vanish condition.

## Results

### Data Before Subtracting Results Under the Delayed-Vanish Condition From Those Under the Immediate-Vanish Condition

**Figure 2.**
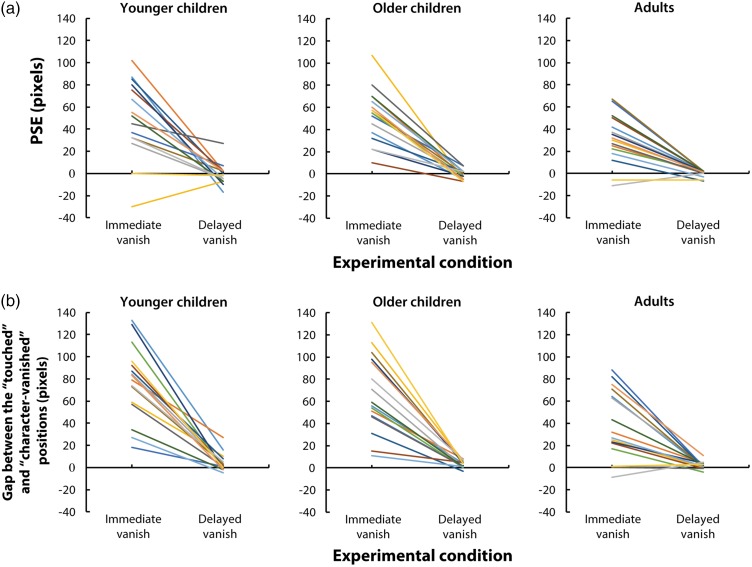
(a) Individual PSEs in the judging task under the immediate-vanish and delayed-vanish conditions. The vertical axes show the mean PSE for each experimental condition. The left, center, and right graphs show the data for younger children, older children, and adults, respectively. The lines in each graph represent individual results. (b) Individual mean displacements between the touched position and the position from which the character vanished in the pointing task under the immediate-vanish and delayed-vanish conditions. The vertical axes show the mean displacement for each experimental condition. The left, center, and right graphs show the data for younger children, older children, and adults, respectively. The lines in each graph represent individual results. Additional individual-level data are available at https://nyu.databrary.org/volume/482. PSE = point of subjective equality.

Although we used the difference between the immediate- and delayed-vanish conditions as a dependent variable (the magnitude of the RM) in the final analysis, we show each mean PSE under the immediate- and delayed-vanish conditions in the judging task in Figure 2(a) and each mean displacement under the immediate- and delayed-vanish conditions in the pointing task in Figure 2(b).

### Main Analysis

**Figure 3.**
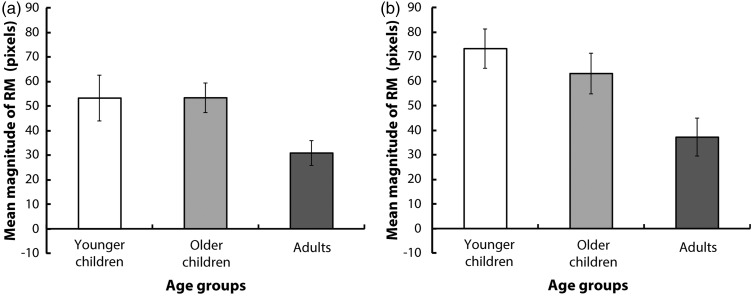
The results of the (a) judging and (b) pointing tasks. The vertical axes show the mean magnitude of RM. The white, light gray, and dark gray bars indicate the mean magnitude of RM in younger children, older children, and adults, respectively. The error bars represent ±1 *SEM*. RM = representational momentum.

Figure 3(a) and (b) presents the results of the judging task and the pointing task, respectively. Because the current two experimental tasks, the judging task and the pointing task, employed different dependent variables (*estimated PSE and gap between the pointed position and the position where the target vanished*, respectively), we first conducted a one-way multivariate analysis of variance (MANOVA) to investigate the effect of age on the magnitude of RM with the mean magnitudes of RM in the judging and pointing tasks as dependent measures. The MANOVA revealed that the effect of age was significant (Wilks’λ = 0.775, *p* = .023, multivariate η^2 ^= 0.120). An additional one-way analysis of variance (ANOVA) for each of the two dependent measures (RM in the judging and pointing tasks) indicated that the main effect of age was significant in both the judging and pointing tasks, *F*(2, 45) = 3.387, *p* = .043, η_p_^2 ^= 0.131, and *F*(2, 45) = 5.379, *p* = .008, η_p_^2 ^= 0.193, respectively. These results indicate again that the magnitude of RM decreased with age in the current study. On the other hand, the post hoc multiple comparisons (Tukey’s HSD tests, α = 0.05) for the one-way ANOVAs revealed that the difference between any pair of the three age groups was not significant in the judging task (younger [*M* = 53.25, *SE* = 9.29] vs. older children [*M* = 53.38, *SE* = 6.08]; younger children vs. adults [*M* = 30.88, *SE* = 5.03]; older children vs. adults), whereas the difference between the younger children (*M* = 73.31, *SE* = 8.02) and the adults (*M* = 37.19, *SE* = 7.70) was significant in the pointing task. The differences between the younger and older children (*M* = 63.13, *SE* = 8.36) and between the older children and the adults were not significant in the pointing task. The results of the post hoc comparisons implied that the age difference in the magnitude of RM was relatively modest in the judging task.

## Discussion

The significant main effect of age on the magnitude of RM revealed by the MANOVA indicates that RM magnitude decreased significantly with age in the current study. This trend was mostly replicated by further analysis with a separate one-way ANOVA for each of the two dependent variables; the main effect of age was significant in both the judging and pointing tasks. Thus, the main conclusion of the current study is that the magnitude of RM decreases with age as reported by Hubbard et al. (1999).

Post hoc multiple comparisons (Tukey’s HSD test) performed on the results of the separate one-way ANOVA for each of the judging and pointing tasks revealed a significant difference in the magnitude of RM between younger children and adults in the pointing task, whereas the post hoc comparison revealed no significant difference in the magnitude of RM between any pair of the three age groups in the judging condition. These results suggest that the age effect on the magnitude of RM was weak in the judging task. The results of the judging task are similar to those reported by Futterweit and Beilin (1994), who found no significant difference in the RM magnitude between younger or older children and adults. The modest age effect on RM in the current judging task might be due to the small effect size of developmental change in the magnitude of RM measured by the current judging task. For instance, the effect size of the main effect of age in the one-way ANOVA was smaller for the judging task (η_p_^2 ^= 0.131) than for the pointing task (η_p_^2 ^= 0.193). The relatively small effect size might result in weaker statistical power such that it was harder for some statistical tests (e.g., the multiple comparisons in the current study) to detect a significant age effect in the judging task than the pointing task (although the age effect on RM may actually exist under both the judging and pointing conditions). It is unclear whether a difference in the tasks (e.g., judging task vs. pointing task) generally affect the effect size during a developmental change in RM. However, the inconsistency between Futterweit and Beilin (1994) and Hubbard et al. (1999) might be explained by similar reasoning, because the effect size of developmental change in RM tends to be smaller with judging tasks (e.g., Futterweit & Beilin, 1994; the current judging task) than with pointing tasks (e.g., Hubbard et al., 1999; the current pointing task), it may be more difficult to detect a significant age difference in RM with judging tasks than with pointing tasks. The impact of more systematic relationships between differences in experimental tasks and effect sizes on age-related changes in RM should be examined in future investigations.

Another possible explanation for the inconsistency between Futterweit and Beilin (1994) and Hubbard et al. (1999) is the difference in the mean age of their participants. The mean age of younger children was 8.9 years (range = 8 months) and that of the older children was 10.9 years (range = 6 months) in Futterweit and Beilin (1994), whereas the mean age of younger children was 6.7 years (range = 3.4 years) and that of the older children was 10.7 years (range = 3.9 years) in Hubbard et al. (1999). The younger children in Futterweit and Beilin (1994) were about 2 years older than the younger children in Hubbard et al. (1999). The 2-year difference in mean age might have contributed to the inconsistency between the two studies. It is plausible that the larger magnitude of RM in children than adults might be typical for relatively young children (e.g., until 6–7 years) but not for older (e.g., >8–9 years) children. However, because the age ranges of the children were not matched between Futterweit and Beilin (1994) and Hubbard et al. (1999), it is difficult to directly compare the mean ages of children in these two previous studies. For example, Hubbard et al. (1999) reported that the age range of the younger children who participated in their study was 5.3 to 8.6 years. This means that the age range of the younger children in Futterweit and Beilin (1994) and that in Hubbard et al. (1999) partially overlapped (note that the same thing is also applicable to the comparison between the current study [younger children: *M* = 7.4 years, range = 6.7–9.1 years; older children: *M* = 10.8 years, range = 9.8–12.0 years] and Futterweit & Beilin, 1994). Hence, although the difference in the mean age of the participants might have contributed to the inconsistency between Futterweit and Beilin (1994) and Hubbard et al. (1999; or the current study), the difference in mean age does not explain all the inconsistencies among these studies.

It should be noted that the current pointing task and the task used by Hubbard et al. (1999) are not identical; the current task involved touching the vanishing point on the touch screen with one’s hand, whereas the task used by Hubbard et al. (1999) involved pointing to a vanishing point on a computer screen with a cursor moved by handling a computer mouse. Thus, despite previous research on pictorial illusions (e.g., Aglioti et al., 1995; Haffenden & Goodale, 1998, 2000; Haffenden et al., 2001), the conclusion that the difference in RM between the judging and pointing tasks was related to a distinction between *seeing* and *acting* should not be generalized to current and previous results regarding the development of RM without caution.

The aim of this study was to examine whether the difference in the experimental tasks would explain previously reported inconsistencies in the development of RM; therefore, we did not directly address the reason for the difference in RM magnitude between young children and adults in the pointing task. One may speculate that RM is more pronounced in younger children than in adults because younger children have more difficulty controlling their motor actions than do adults during the pointing task. For instance, when young children (6–8 years) pointed at a static target, they tended to overshoot the target location (i.e., when they pointed at a target on the right or left, they tended to overshoot to the right or left of the target), and the tendency to overshoot was more pronounced under conditions in which a target appeared in the right visual field (Pellizzer & Hauert, 1996). This suggests that the presumed RM observed among children in the current pointing task might not actually constitute RM and may reflect only overshooting when pointing to a target position (i.e., biomechanical constraints). However, in the current study, the magnitude of RM was calculated by subtracting the results obtained from the trials using the delayed-vanish condition from those obtained from trials with the immediate-vanish condition. It should be noted that there is no a priori reason to infer that the magnitude of the overshooting in the pointing action would differ between the two conditions due to any causal factor other than RM. In other words, the magnitude of RM calculated by subtracting the results of the delayed-vanish condition from those of the immediate-vanish condition should represent overshooting caused by RM; thus, the subtraction method enabled us to compensate for potential biases arising from the motor abilities of any age-group.

## Note

1 Although the authors performed pilot observations and confirmed that a visual stimulus with the maximum speed produces RM, the maximum speed of the character in the current study (52.1 deg/s) was relatively faster than that typically adopted by most studies of RM. As far as we know, there has been no empirical evidence showing that children aged between 6 and 12 years are unable to detect or perceive directional motion at a speed of at least 52.1 deg/s. However, the sensitivity for detecting or perceiving such high-speed directional motion may differ between children and adults. It will be important to test whether the development of RM differs according to the speed of visual stimuli.
